# Effects of different tillage systems and mowing time on nutrient accumulation and forage nutritive value of *Cyperus esculentus*

**DOI:** 10.3389/fpls.2023.1162572

**Published:** 2023-04-14

**Authors:** Yi Du, Yulin Zhang, Xutian Chai, Xiangyi Li, Abd Ullah, Waqar Islam, Zhihao Zhang, Fanjiang Zeng

**Affiliations:** ^1^ State Key Laboratory of Desert and Oasis Ecology, Xinjiang Institute of Ecology and Geography, Chinese Academy of Sciences, Urumqi, China; ^2^ Xinjiang Key Laboratory of Desert Plant Roots Ecology and Vegetation Restoration, Xinjiang Institute of Ecology and Geography, Chinese Academy of Sciences, Urumqi, China; ^3^ Cele National Station of Observation and Research for Desert-Grassland Ecosystems, Cele, China; ^4^ University of Chinese Academy of Sciences, Beijing, China; ^5^ College of Ecology and Environmental, Xinjiang University, Urumqi, China

**Keywords:** continuous/rotation cropping, mowing time, relative abundance, nutrient content, structural equation model

## Abstract

Revealing the complex relationships between management practices, crop growth, forage nutritive value and soil quality will facilitate the development of more sustainable agricultural and livestock production systems. *Cyperus esculentus* is known as the king of oil crops and high-quality forage. However, there is little information about the effects of different planting modes {continuous cropping (CC)/rotation cropping (RC)} and initial mowing time on the plant nutrient accumulation and forage nutritive value. Here, in a field experiment, we designed two planting patterns, *C. esculentus* CC and *C. esculentus* - wheat RC. The leaves, tubers, roots, and soil samples were collected at three mowing time (on the 78^th^, 101^th^, and 124^th^ days after seed sowing). Results revealed that RC significantly increased the total nitrogen (TN) and potassium (TK) content of the tuber (*p*<0.05), while significantly decreased the TN, total phosphorus (TP), crude protein (CP), and acid detergent fiber (ADF) contents of the leaves. Under the CC pattern, the TN, TP, and TK content of roots increased significantly on the 78^th^ days after seed sowing, and the TK content of tubers increased significantly. Under the RC pattern, the ether extract (EE) content of tubers increased significantly on the 124^th^ days after seed sowing, while the CP and TN content of leaves decreased significantly. Correlation analysis showed that soil pH was negatively correlated with TN content in leaves, tubers, and roots. The structural equation model showed that the soil pH directly affected the plant nutrient accumulation and forage nutritive value (*β*=0.68) *via* regulating these properties by changing soil available nutrients, anions, cations, and total nutrients. Overall, we propose that RC for *C. esculentus*-wheat is should not be recommended to maximize tubers and forage yield.

## 1 Introduction

Continuous cropping (CC) is the practice of growing the same crop on the same land for several years. This practice usually leads to the loss of soil nutrients and aggravation of pests and diseases, ultimately causing the decline in crop yield and quality (Xiong et al., 2015; Delang, 2018). In contrast, rotation cropping (RC), the scientific and orderly planting of different crops in the same field can improve the water use efficiency of crops (Zhou et al., 2011; Li et al., 2014; Peterson et al., 2020), balance soil nutrients, enhance soil functions, and increase crop yield (Larkin, 2003; Zhou et al., 2011; Moulin et al., 2015). However, the advantages of crop rotation are influenced by crop type, soil moisture and nutrient availability (Giacometti et al., 2021). For example, compared with non-leguminous crops, rotation with leguminous crops can effectively improve soil nitrogen (N) availability and system productivity (Ilyas et al., 2022). However, since different crops have different soil requirements (e.g. nutrient requirements), long-term rotation can accelerate soil nutrient loss and can intensify nutrient competition between crops and microorganisms, which adversely affects the yield of the two crops in rotation (Brankatschk and Finkbeiner, 2015). Therefore, accurate assessment of the response of specific crops to CC and RC is crucial for farmers to choose suitable cropping strategies in terms of soil fertility and nutrients of different organs.

Crude protein (CP), ether extract (EE), acid detergent fiber (ADF), and neutral detergent fiber (NDF) are important indexes for evaluating forage nutritive value (Richman et al., 2015). Some studies have shown that the soluble sugar (SS) content in the rotation was significantly higher than in CC (Yang et al., 2010; Hou et al., 2013). Meanwhile, the research found that the RC significantly increased the contents of CP and EE, reduced the content of SS, and improved the quality of tubers (Ma and Han, 2000; Wang et al., 2017). Mowing is a common practice to increase forage efficiency and yield for livestock (Zhao et al., 2021). The effects of initial mowing time on grassland productivity have been extensively studied under different climatic and soil conditions (Donaghy et al., 2008; Binnie et al., 2010). For example, medium initial mowing (15 July to 15 August) increased total dry matter yield and nutritional value of *Leymus chinensis* (Trin.) Tzvel. in Songnen grassland, China (Zhao et al., 2021). On the contrary, the early (April) and late (May) mowing reduced the total yield of the forage sugarcane in the Amami Region of Kagoshima Prefecture, Japan (Sakaigaichi et al., 2010). These inconsistencies in forage yield and forage nutritive value were mainly due to the interaction of initial mowing time, grass species and geographical location.

Animal husbandry is a mainstay of economic development in arid and semi-arid regions, however it is suffering from inadequate forage production and supply due to water constraints (Blum, 2009; Jia et al., 2022). Therefore, it is of great significance to select crops that are more suitable for both forage yield, grain and oil supply under water shortage conditions to improve livestock production and may alleviate food crisis in arid and semi-arid areas. Meanwhile, the *Cyperus esculentus* is a grass like plant widely distributed in many northern temperate regions and is gaining popularity in China, India, Egypt and the United States as an energy crop (Arafat et al., 2009; Bamishaiye et al., 2010; De Castro et al., 2015; Ayeni, 2022). Its aboveground parts (leaves) are used as forage, and its belowground parts are edible tubers with sweet taste with high levels of protein, fatty acids and starch, that make it a potential alternative to wheat and soybeans in many countries (Sánchez-Zapata et al., 2012; Ahmed and Hussein, 2014; Codina-Torrella et al., 2015; Ayeni, 2022). However, to our best knowledge, there is a little information on the nutritive value of the above- and under-ground parts of *C. esculentus* in respond to different planting modes (CC vs RC) and initial mowing time, even though it has been cultivated for many years in the desert oasis transition zone (Tan et al., 2022; Zhang et al., 2022).

Therefore, to research the understanding of response mechanisms to planting modes in *C. esculentus* is timely. Herein, we designed a field study containing two dependent treatments, two planting modes (CC vs RC) and three initial mowing time (on the 78^th^, 101^th^, and 124^th^ days after seed sowing). Total nitrogen (TN), total phosphorus (TP), and total potassium (TK) contents in leaves, tubers, and roots were used to evaluate the nutrients of *C. esculentus*, CP, EE, and NDF contents in leaves, and EE, CP, and ADF in tubers were determined to characterize the above- and belowground nutritive value of *C. esculentus*, respectively. Our objectives were to (i) elucidate the influences of planting modes and initial mowing time on nutrient content and forage nutritive value of *C. esculentus*, and (ii) identify the factors affecting the nutrient content and forage nutritive value of *C. esculentus*. These attempts will provide a reference for the management of *C. esculentus* in the desert oasis transition zone.

## 2 Material and methods

### 2.1 Site description and experiment design

This experiment was carried out at Shache farm in Xinjiang (38.41° N, 77.24° E). The region has a warm temperate continental climate. The mean annual temperature, precipitation, and evaporation are 11.6°C, 53.3 mm, and 2246 mm, respectively. The frost-free period is 225 days and average temperature is above 10°C for the whole year (Arzigul and Xianmixinuer, 2017).

Our experiment was established on May 2018. The background of soil physicochemical properties in both CC and RC plots was similar, pH 8.87, soil organic matter (SOM) 2.527 g·kg^-1^, TN 0.093 g·kg^-1^, TP 0.325 g·kg^-1^, TK 16.133 g·kg^-1^, available nitrogen (AN) 18.677 mg·kg^-1^, the available phosphorus (AP) 4.260 mg·kg^-1^, the available potassium (AK) 50.867 mg·kg^-1^. After wheat harvest in May, the seeds of “fengchan No. 2”, a variety of *C. esculentus*, were sown simultaneously in the CC and RC treatments at the end of May by using a drop planter using 75 kg (seed)·hm^-2^ with the density of 20 × 35 cm (plant spacing × row spacing). The planting density was 142,860 plants·hm^-2^. The N, P, K fertilizer were dissolved in water and dripped to the root during the whole growth period every year. In each year, the first time of drip irrigation was carried out on the 30^th^ day after the emergence of seedlings, and then six times at 15-day intervals. Before the final harvest (end of September) every year, the dosage of water and fertilizer was 6500 m^3^·hm^-2^ water, 335 kg·hm^-2^ N-fertilizer (urea, 46% N), 218 kg·hm^-2^ P-fertilizer (monoammonium phosphate, 10% N; 50% P_2_O_5_), and 68 kg·hm^-2^ K- fertilizer (potassium sulphate, 50% K_2_O).

Our sampling work was conducted on the 78^th^ day after seed sowing. In each planting mode, three 10 × 5 m plots, 5 m apart, were set at each mowing time (on the 78^th^, 101^th^, and 124^th^ days after seed sowing). In each plots, three 50 × 50 cm quadrats along the diagonal were set to collect plant (leaves, roots, and tubers) samples. Five topsoil (0~30 cm) samples along the diagonal in each plots were collected and mixed to prepare one sample to determine the soil physicochemical properties.

### 2.2 Measurement of plant nutrient content

The leaves, tubers, and roots were washed with running water and dried in an oven (65°C) until the constant weight was used to obtain biomass. These dry matters were powdered in a vibratory disc mill (RS200, Retsch GmbH Inc., Haan, Germany). To subsequent determination of nutrient content and quality of different organs after passing through a 0.42 mm sieve. The TN was measured using an elemental analyzer (2400 II CHN elemental analyzer; Perkin-Elmer, USA), TP was determined using the molybdenum-antimony anti-spectrophotometric method, and TK was determined by flame photometry (Bao, 2000). The EE was extracted by the Soxhlet extractor method, 2~5 g sample was weighed, adding anhydrous ether or petroleum ether was to 2/3 of the bottle content volume, heated in a water bath, so that ether or petroleum ether was continuously refluxed and extracted for 6~12 h. The CP content was determined by the Kjeldahl nitrogen determination method, 2~5g samples were weighed, and 0.5 g copper sulfate and 10 mL sulfuric acid were added. The content of ADF and NDF was determined by Fan’s detergent fiber analysis method (Li, 2000). 2g samples were accurately weighed and divided into two parts, one of which was added with 100 mL neutral detergent. Another 1 g sample was added with 100 mL acid detergent. Boil in 5~10 min, and keep boiling for 60 min. After boiling, remove the straight beaker, pour the solution in the beaker into the known weight glass heap installed on the filter bottle for filtration, remove all the residue in the beaker, and rinse the glass heap and residue with boiling water until the filtrate is neutral. Rinse twice with 20 mL acetone, and suction filtration. The glass pile was placed in a 105°C oven for 2 h and then cooled in a dryer for 30 min.

### 2.3 Measurement of soil physicochemical properties

Soil samples were air-dried after the removal of roots. TN, TP, TK, AN, AP, AK, and pH were determined after passing through a 2 mm sieve. The value of pH was evaluated by a PHS-3C digital pH meter in a 1:5 soil-water ratio suspension (IQ 150, IQ Scientific Instruments, CA, USA). The TN content was determined by using the CuSO_4_–Se powder diffusion method (Hooper and Vitousek, 1998). The Mo-Sb and Van-Mo-yellow colorimetry methods were applied to detect TP content (Lu et al., 2015). The TK content was determined by using molybdenum‐antimony colorimetry (Lu et al., 2015). Additionally, soil available N was tested using 2‐M KCl extracts by AA3‐automated flow injection analysis (Bran + Luebbe GmbH, Norderstedt, Germany); available P was extracted using 0.5 M NaHCO_3_, and available K was measured by the NH4OAc method (Neff et al., 2005; Warra et al., 2015). Soil organic matter (SOM) was determined by the wetoxidation technique. Total salt (TS) content and salt ion content were extracted with soil extract (soil and water ratio 5:1). The TS content was determined by using the conductivity method. The HCO^3-^, Cl^-^, SO_4_^2-^, Ca^2+^, Mg^2+^, K^+^, and Na^+^ contents were measured according to the mothed of Bao (2000) and Lu (1999). Ca^2+^, Mg^2+^, K^+^, and Na^+^ were determined by plasma spectrometer with the test solution prepared by three acid mixture (1 mL 60% HClO_4_, 6 mL HNO_3_) digestion method (IRIS-ICP, TJA Ltd, USA). HCO^3-^, Cl^-^, and SO_4_^2-^ were determined by chemical titration.

### 2.4 Statistical analyses

All statistical analyses were conducted in the R v4.1.0 (R Core Team, 2018). Most of the results were visualized using the ‘ggplot2 package’. Two-way ANOVA was used to analyze the independent and interaction effects of CC/RC and mowing time on soil properties and nutrient content and forage nutritive value of *C. esculentus*. Duncan’s method was used for multiple comparisons (α=0.05), and the significance level was 0.05, expressed as mean ± standard error (SE, n = 3). T-test was used to analyze and compare the differences between different tillage patterns (CC and RC) at the same mowing time. Correlation analysis of soil properties, nutrient content, and forage nutritive value was computed and visualized by using the ‘psych package’ and ‘corrplot package’. The causal relationships between soil properties, nutrient content and forage nutritive value of *C. esculentus* were explored by the structural equation model using Amos-24 software.

## 3 Results

### 3.1 Soil physicochemical properties

The pH value, SOM, TK, and AP contents in soil were independently influenced by both planting pattern (CC/RC) and mowing time, while the AN and AK contents were independently influenced by mowing time and planting pattern, respectively (*p*<0.05, **Table 1**). The interaction of these factors significantly changed TK content (*p*<0.05). However, soil TN and TP contents were not significantly affected by planting mode and mowing time (*p*>0.05). The RC mode significantly increased soil pH, while significantly reduced SOM, TK, AP, and AK contents (*p*<0.05, **Table 2**). With the postponement of mowing time, soil pH gradually increased, while SOM and AP gradually decreased. The AN content showed a significant decline at the end of mowing time (**Table 2**). Except for Cl^-^ and SO2-4 that reduced in RC mode (**Table 3**), the other parameters of soil salinity did not respond significantly to planting pattern and mowing time (**Table 4**).

**Table 1:** Two-way ANOVA analysis of effects of the planting mode and mowing time on soil physicochemical properties.

Factor	pH	SOM (g•kg^-1^)	TN (g•kg^-1^)	TP (g•kg^-1^)	TK (g•kg^-1^)	AN (mg•g^-1^)	AP (mg•g^-1^)	AK (mg•g^-1^)
planting mode (CC/RC)	6.09*	5.13*	2.74	0.45	13.79**	0.05	5.83*	10.81**
Mowing time (M)	4.86*	4.04*	0.93	3.50	68.61***	4.62*	4.20*	0.33
CC/RC×M	0.84	1.51	2.71	1.06	5.70*	0.98	0.77	0.16

Here: CC, continuous cropping; RC, rotation cropping. M, Mowing time (T1, on the 78^th^ day after seed sowing; T2, on the 101^th^ day after seed sowing; T3, on the 124^th^ day after seed sowing). SOM, soil organic matter; TN, total nitrogen; TP, total phosphorus; TK, total potassium; AN, available nitrogen; AP, the available phosphorus; AK, the available potassium. Values indicate results of F value *p < 0.05; **p < 0.01; ***p < 0.001. The same as below.

**Table 2:** Soil physical and nutrient content.

Planting mode	Mowing time	pH	SOM (g•kg^-1^)	TN (g•kg^-1^)	TP (g•kg^-1^)	TK (g•kg^-1^)	AN (mg•g^-1^)	AP (mg•g^-1^)	AK (mg•g^-1^)
	T1	8.56 ± 0.09 a	2.24 ± 0.14 a	0.10 ± 0.11 a	0.35 ± 0.006 a	15.93 ± 0.15 b	61.07 ± 24.86 a	9.72 ± 0.81 a	94.70 ± 23.05 a
CC	T2	8.83 ± 0.04 a	1.84 ± 0.15 a	0.08 ± 0.01a	0.29 ± 0.008 a	15.20 ± 0.10 c	51.90 ± 14.37 a	5.65 ± 2.58 a	94.43 ± 3.66 a
	T3	8.83 ± 0.09 a	1.85 ± 0.04 a	0.08 ± 0.002 a	0.33 ± 0.04 a	16.43 ± 0.09 a	18.53 ± 7.60 a	5.04 ± 0.96 a	89.40 ± 6.79 a
	T1	8.81 ± 0.11 a	1.86 ± 0.04 a	0.07 ± 0.004 a	0.36 ± 0.01 a	15.37 ± 0.29 b	45.17 ± 12.64 ab	5.54 ± 0.59 a	65.03 ± 5.45 a
RC	T2	8.88 ± 0.02 a	1.71 ± 0.09 a	0.07 ± 0.006 a	0.33 ± 0.02 a	14.33 ± 0.07 c	82.57 ± 27.83a	4.01 ± 0.23 ab	60.33 ± 1.92 a
	T3	8.98 ± 0.06 a	1.81 ± 0.09 a	0.09 ± 0.01 a	0.31 ± 0.01 a	16.53 ± 0.03 a	13.23 ± 3.51 b	3.59 ± 0.68 b	67.10 ± 7.81 a

Here: CC, continuous cropping; RC, rotation cropping. M, Mowing time (T1, on the 78^th^ day after seed sowing; T2, on the 101^th^ day after seed sowing; T3, on the 124^th^ day after seed sowing). Different lowercase letters (a, b, and c) indicate that the same farming mode and different mowing time have significant differences (Duncan’s test, p<0.05). The same as below.

**Table 3:** saltiness of soil.

Planting mode	Mowing time	Cl^-^ (mg•g^-1^)	SO_4_^2-^ (mg•g^-1^)	Ca^2+^ (mg•g^-1^)	K^+^ (mg•g^-1^)	Mg^2+^ (mg•g^-1^)	Na^+^ (mg•g^-1^)	HCO_3_^-^ (mg•g^-1^)	TS (g•kg^-1^)
	T1	0.08 ± 0.02 a	0.33 ± 0.07 a	0.64 ± 0.41 a	0.12 ± 0.10 a	0.11 ± 0.07 a	0.14 ± 0.08 a	0.09 ± 0.01 a	1.94 ± 0.80 a
CC	T2	0.08 ± 0.03 a	0.23 ± 0.03 a	0.12 ± 0.01 a	0.03 ± 0.001 a	0.02 ± 0.003 a	0.07 ± 0.02 a	0.10 ± 0.002 a	0.76 ± 0.14 a
	T3	0.11 ± 0.04 a	0.39 ± 0.14 a	0.20 ± 0.05 a	0.03 ± 0.005 a	0.04 ± 0.01 a	0.10 ± 0.03 a	0.09 ± 0.008 a	1.11 ± 0.33 a
	T1	0.04 ± 0.01 a	0.16 ± 0.04 a	0.46 ± 0.27a	0.16 ± 0.14 a	0.08 ± 0.07 a	0.36 ± 0.32 a	0.09 ± 0.01 a	1.71 ± 1.00 a
RC	T2	0.03 ± 0.01 a	0.13 ± 0.01 a	0.11 ± 0.01 a	0.01 ± 0.0005 a	0.02 ± 0.001 a	0.05 ± 0.005a	0.10 ± 0.003 a	0.52 ± 0.06 a
	T3	0.06 ± 0.02 a	0.22 ± 0.07 a	0.13 ± 0.03 a	0.02 ± 0.003 a	0.02 ± 0.003 a	0.06 ± 0.02 a	0.09 ± 0.003 a	1.00 ± 0.07 a

Here: CC, continuous cropping; RC, rotation cropping. M, Mowing time (T1, on the 78^th^ day after seed sowing; T2, on the 101^th^ day after seed sowing; T3, on the 124^th^ day after seed sowing).

**Table 4:** Two-way ANOVA analysis of effects of the planting mode and mowing time on soil physicochemical properties.

Factor	Cl^-^ (mg•g^-1^)	SO_4_^2-^(mg•g^-1^)	Ca^2+^ (mg•g^-1^)	K^+^ (mg•g^-1^)	Mg^2+^ (mg•g^-1^)	Na^+^ (mg•g^-1^)	HCO_3_^-^(mg•g^-1^)	TS (g•kg^-1^)
planting mode (CC/RC)	6.16*	6.17*	0.29	0.00	0.29	0.22	0.01	0.21
Mowing time (M)	0.93	1.57	2.78	1.93	2.15	1.21	0.69	2.46
CC/RC×M	0.01	0.17	0.10	0.07	0.03	0.53	0.09	0.11

Here: CC, continuous cropping; RC, rotation cropping. M, Mowing time (T1, on the 78^th^ day after seed sowing; T2, on the 101^th^ day after seed sowing; T3, on the 124^th^ day after seed sowing). TS. Total salt; Values indicate results of F value *p < 0.05.

### 3.2 The nutrient content and allocation of the leaves, tubers, and roots

**Figure 1:**
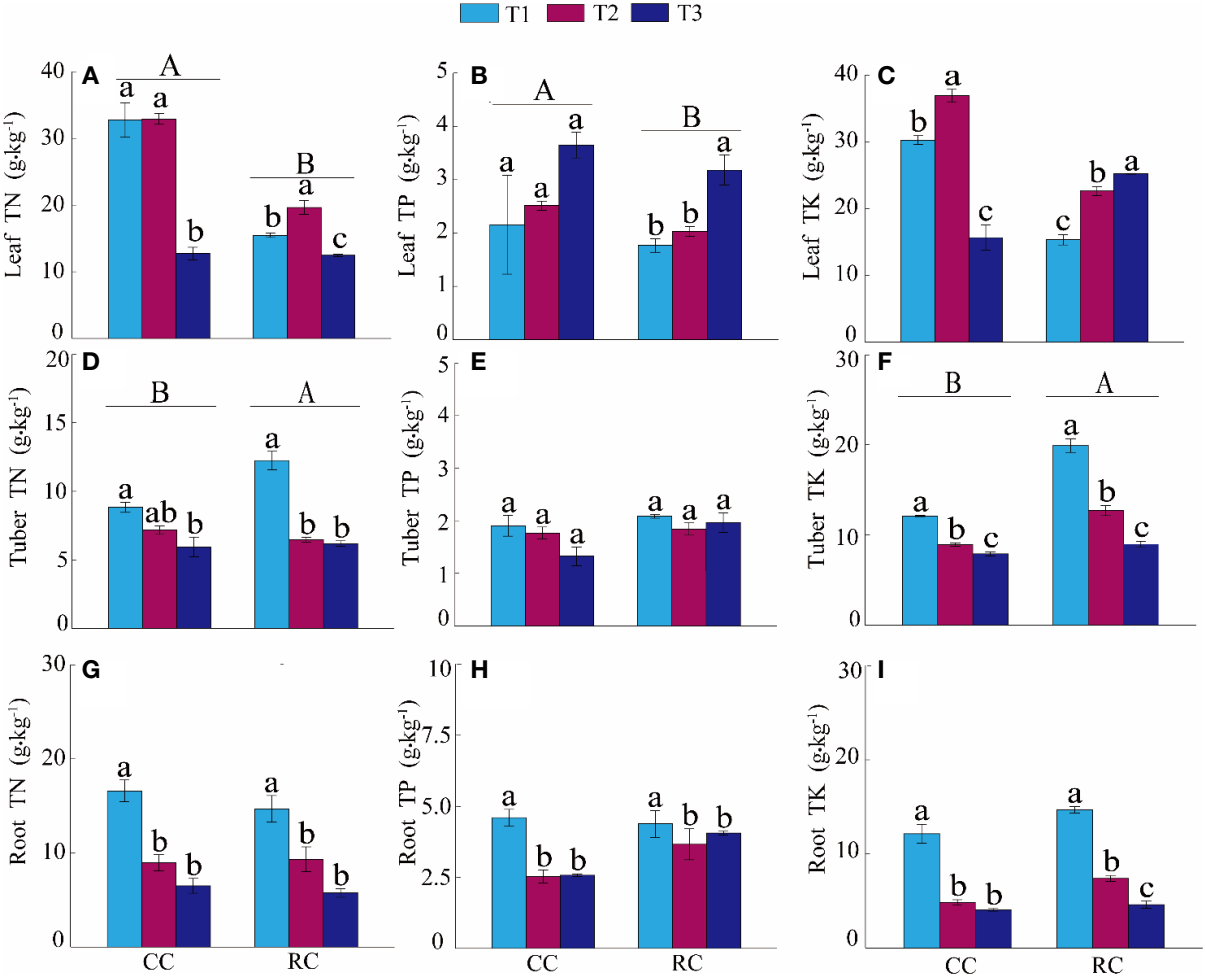
Effects of the continuous cropping (CC)/rotation cropping (RC) and mowing time on [**A** (total nitrogen (TN)], **B** [total phosphorus (TP)], and **C** [total potassium (TK)] of the leaves, [ **D** (total nitrogen (TN)], **E** [total phosphorus (TP)], and **F** [total potassium (TK)] of the tubers, and [ **G** (total nitrogen (TN)], **H** [total phosphorus (TP)], and **I** [total potassium (TK)] of the roots.

Planting mode and mowing time independently and interactively impacted the TN and TP contents in leaves and tubers, that is, the response of these two elements in the specific organ to mowing times varied from CC and RC systems (*p*<0.05, **Table 5**). For example, RC significantly decreased foliar N and P content, while these two elements in tubers were improve in CC systems (*p*<0.05, **Figure 1**). No interaction between planting mode and mowing time on nutrient was observed in roots (**Table 5**; **Figure 1**).

**Table 5:** Two-way ANOVA analysis of effects of the planting mode and mowing time on the physicochemical property of the leaves, tubers, and roots of *C. esculentus*.

Factor	Leaves			Tubers			Roots		
	TN (g•kg^-1^)	TP (g•kg^-1^)	TK (g•kg^-1^)	TN (g•kg^-1^)	TP (g•kg^-1^)	TK (g•kg^-1^)	TN (g•kg^-1^)	TP (g•kg^-1^)	TK (g•kg^-1^)
planting mode (CC/RC)	100.93***	1.75	64.52***	6.91*	6.06*	147.99***	0.79	8.79*	21.49**
Mowing time (M)	68.89***	6.83**	47.18***	54.07***	2.74	166.20***	43.27***	10.33**	188.38***
CC/RC×M	25.22***	0.01	97.49***	10.90**	2.03	31.99***	0.59	3.74	2.64

Here: CC, continuous cropping; RC, rotation cropping. M, Mowing times (T1, on the 78^th^ day after seed sowing; T2, on the 101^th^ day after seed sowing; T3, on the 124^th^ day after seed sowing). Values indicate results of F value.

*p < 0.05; **p < 0.01; ***p < 0.001.

### 3.3 The forage nutritive value of the leaves, tubers, and roots

**Figure 2:**
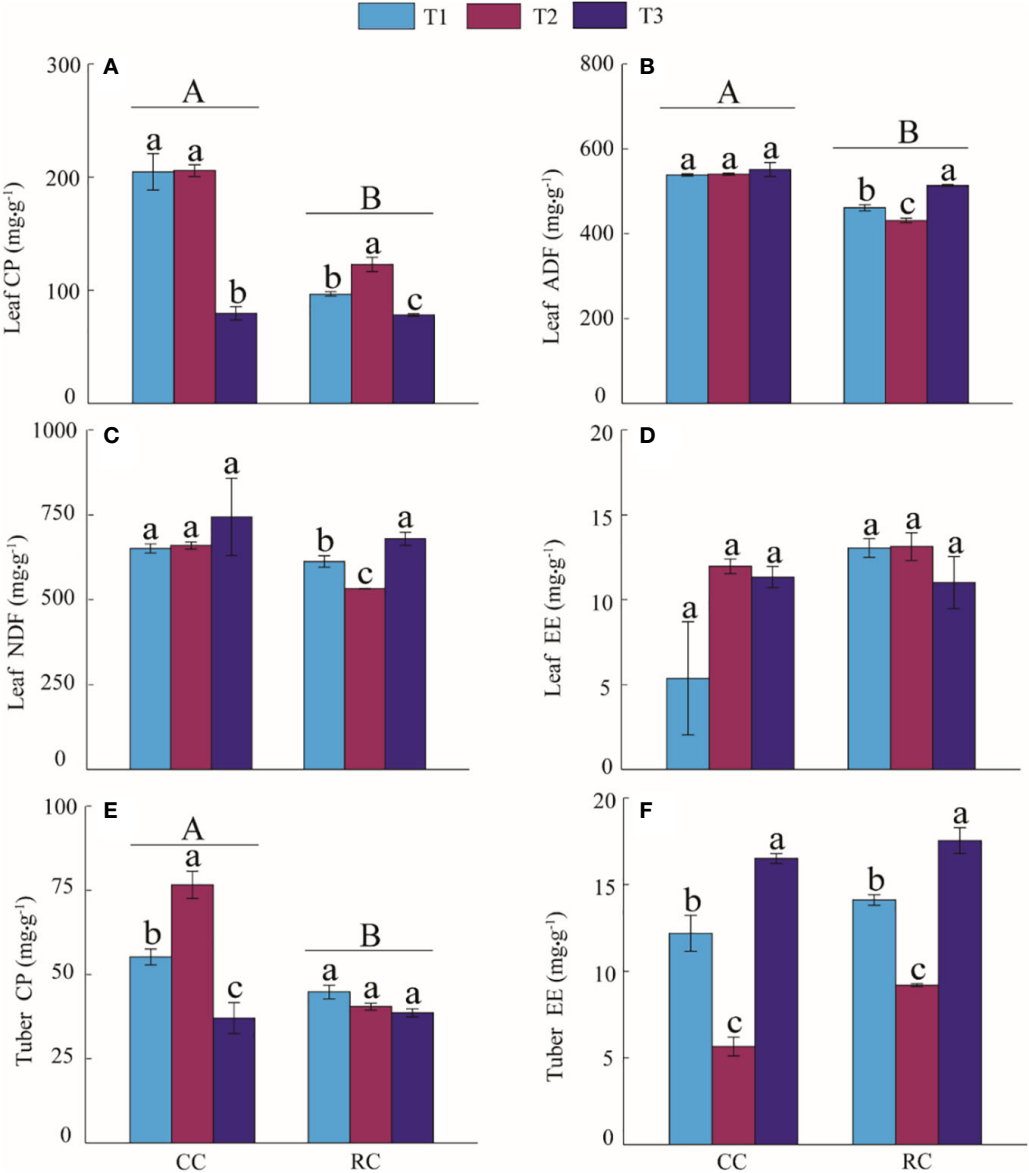
Effects of the continuous cropping (CC)/rotation cropping (RC) and mowing time on [**A** (crude protein (CP)], **B** [acid detergent fiber (ADF)], **C** [neutral detergent fiber (NDF)], and **D** [ether extract (EE)], contents of the leaves and **E** [crude protein (CP)] and **F** [ether extract (EE)], contents of the tubers.

Planting mode interacted with mowing time significantly altered the foliar CP and NDF contents and the CP contents in tubers (*p*<0.05, **Table 6**). Foliar ADF content was significantly influenced by planting modes. Planting modes and mowing time independently affected EE content in tubers, while they did not exert significant effects on foliar EE content (*p*>0.05). Compared with CC system, RC had a significantly adverse effect on the accumulation of CP and ADF in leaves, and CP in tubers (**Figures 2A, B, E**). On the 124^th^ day after seed sowing, more ADF and NDF accumulated in leaves, while the CP contents in leaves and tubers showed a lower level than those at other periods (**Figure 2**).

**Table 6:** Two-way ANOVA analysis of effects of the planting mode and mowing time on the forage nutritive value of the leaves of *C. esculentus*.

Factor	Leaves				Tubers	
	CP (mg•g^-1^)	EE (mg•g^-1^)	NDF (mg•g^-1^)	ADF (mg•g^-1^)	CP (mg•g^-1^)	EE (mg•g^-1^)
planting mode (CC/RC)	101.34***	3.79	137.91***	4.84*	41.02***	20.08**
Mowing time (M)	69.13***	3.01	19.11***	2.26	26.34***	132.80***
CC/RC×M	25.57***	0.49	10.65**	3.63	22.70***	2.29

Here: CC, continuous cropping; RC, rotation cropping. M, Mowing times (T1, on the 78^th^ day after seed sowing; T2, on the 101^th^ day after seed sowing; T3, on the 124^th^ day after seed sowing). Values indicate results of F value.

*p < 0.05; **p < 0.01; ***p < 0.001.

### 3.4 The relationships between the nutrient content and forage nutritive value of leaves, tubers, roots, and soil factors

**Figure 3:**
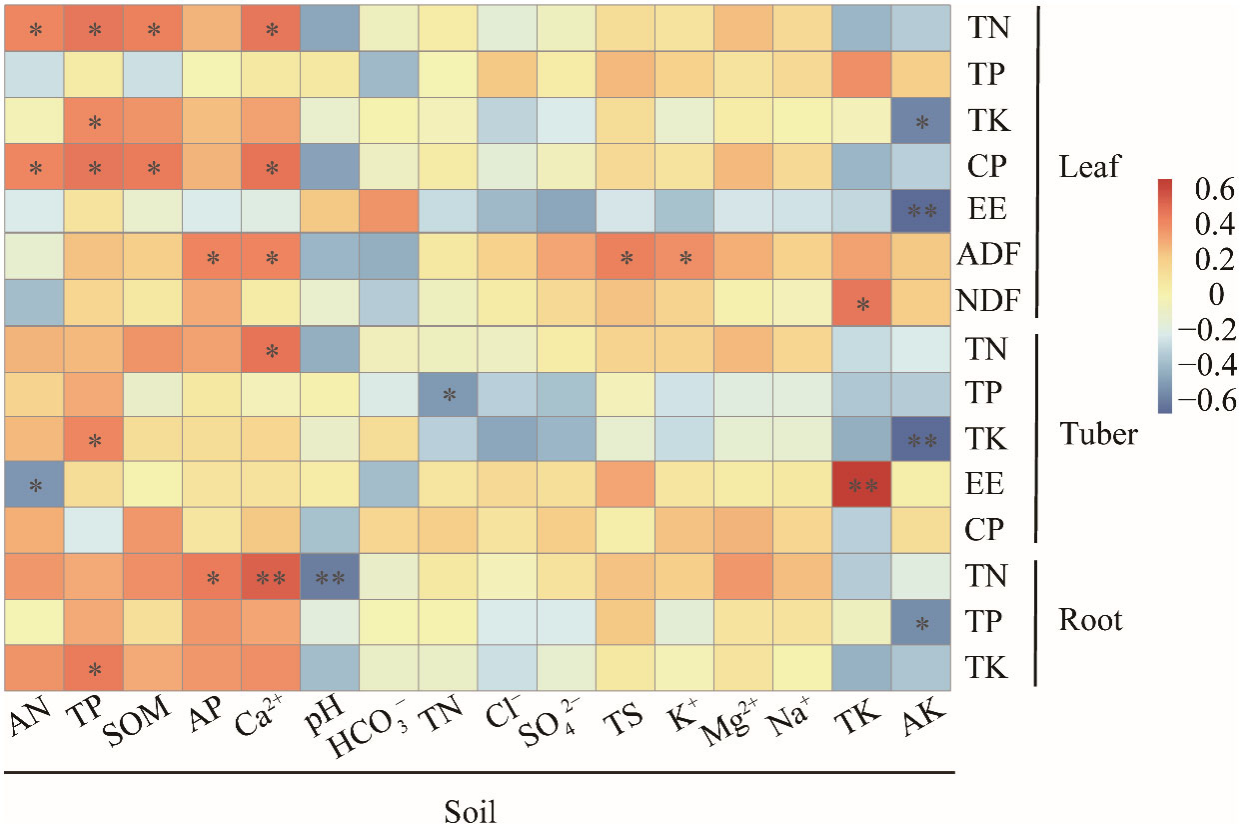
Correlation analysis of leaves, tubers, roots, and soil factors. Here: SOM, soil organic matter; TN, total nitrogen; TP, total phosphorus; TK, total potassium; AN, available nitrogen; AP, the available phosphorus; AK, the available potassium; TS, Total salt; EE, ether extract; CP, Crude protein; ADF, acid detergent fiber; NDF, neutral detergent fiber. *p < 0.05; **p < 0.01.

The Pearson’s correlation analysis showed that foliar forage nutritive value (i.e., CP, EE, ADF, and NDF) had a significant correlation with some soil factors, such as SOM, AN, AP, AK, and Ca^2+^ (*p*<0.05, **Figure 3**). The EE content of tubers was negatively influenced by AN, while positively affected by TK (*p*<0.05). Compared with other elements, plant TP was slightly affected by soil factors, only having a significant positive correlation with TN in the tuber and a significant negative correlation with AK in the root (*p*<0.05).

**Figure 4:**
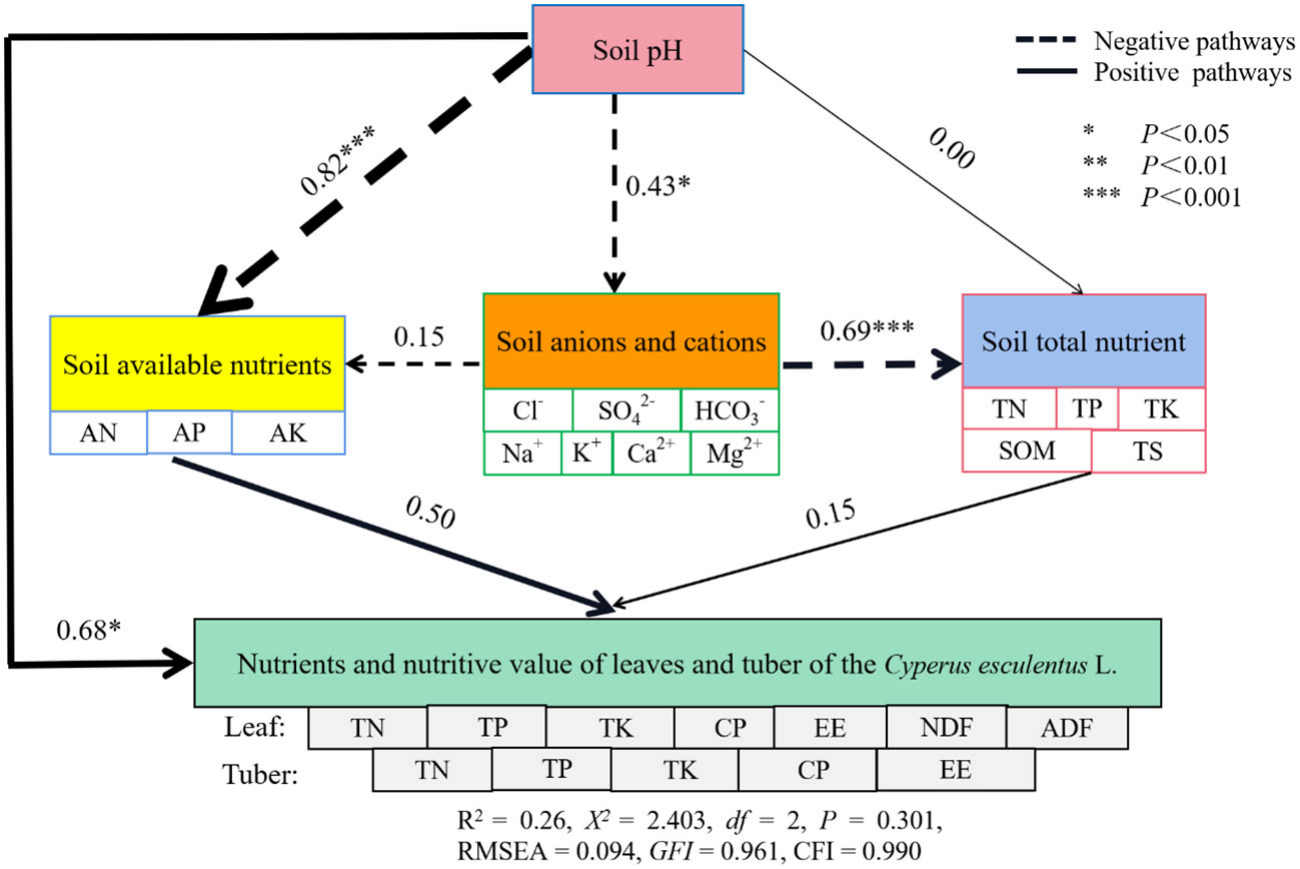
The structural equation model of effects of the continuous cropping (CC)/rotation cropping (RC) on nutrient content and forage nutritive value of leaves and tubers. Here: The principal component (soil available nutrients, soil anions and cations, soil total nutrients, and nutrient and forage nutritive value of the leaves and tubers) first axis data are reported in **Table 7**.

The structural equation model revealed an important role of soil pH in regulating the accumulation of nutrients and forage nutritive value of the leaves and tubers. Soil pH, base ions, cations, and available and total nutrients exhibited 26% of the variation in nutrient and nutritive value of the leaves and tubers (*X^2 =^*2.403, *p*=0.301, **Figure 4**). Among them, the nutrient and nutritive value of leaves and tubers were dominantly and directly affected by soil pH, with a standardized direct effect of 0.68 (**Figure 4**). Additionally, soil pH indirectly affected the nutrients and nutritive value of the leaves and tubers by changing the available nutrients, anions, cations, and total nutrients in soil.

**Table 7:** The principal component first axis data (soil available nutrients, soil anions and cations, soil total nutrients, and nutrient and forage nutritive value of the leaves and tubers).

Soil available nutrients	pH	Soil anions and cations	Soil total nutrient	Nutrients and nutritive value (leaves and tubers)
-0.59267	0.32803	1.54209	-0.98532	-0.54155
-0.64265	0.72498	-0.46442	0.08915	-0.53676
-0.35805	-0.26739	-0.32869	-0.21075	-0.53915
-0.59267	0.32803	1.54209	-0.98532	-0.54155
-0.64265	0.72498	-0.46442	0.08915	-0.53676
-0.35805	-0.26739	-0.32869	-0.21075	-0.53915
0.65725	-1.85517	-0.02982	-0.44283	-0.40714
2.05449	-2.78137	1.5454	-0.81002	-0.82544
0.14463	-0.66433	-0.35341	0.01456	-0.47403
-0.12847	1.25424	-0.54395	0.20505	-0.92046
0.20114	-1.06128	-0.28644	0.28048	-0.7682
-0.1228	-0.59817	1.68642	0.09066	-1.00025
0.82174	-0.46586	-0.1924	0.61666	0.3564
0.33515	0.39419	-0.43289	0.21451	0.49894
0.18698	0.0634	-0.25048	0.20955	0.56515
-0.2876	0.12956	-0.40453	0.48568	0.28125
0.28809	0.26187	-0.48191	0.72617	0.01618
0.02923	0.65882	-0.46377	0.66031	0.20052
0.16063	-0.66433	0.20819	0.04184	0.69623
0.0785	-0.46586	-0.02156	0.09273	0.84186
0.01452	1.18808	-0.46037	-0.11046	1.02772
-0.73504	1.51887	-0.44572	0.15485	0.46659
-0.05167	0.26187	-0.15553	-0.26716	0.53505
-0.46004	1.25424	-0.4152	0.05126	0.5271

## 4 Discussion

In this study, the nutrient and nutritional value of leaves and tubers, as well as the nutrient content of soil were determined and analyzed by selecting different planting patterns (RC and CC) and setting three mowing time. Finally, it was found that RC was not conducive to the growth and development of *C. esculentus*. On the 124^th^ day after seed sowing, the EE content of tubers was increased, which indirectly increased its oil yield.

### 4.1 The effects of RC on plant nutrient accumulation and forage nutritive value

Crop rotation in present study exerted a negative effect of on foliar nutrient (e.g., TN, and TP) accumulation and forage nutritive value of *C. esculentus* (**Figure 1A, B**), inconsistent with the promoting effect of rotation found by previous studies (Sun et al., 2018; Ding et al., 2019; Lenssen et al., 2020). Two possible factors explain this inconsistency. On the one hand, the root distribution of *C. esculentus* and wheat highly overlapped in topsoil (~30 cm depth), which intensifies competition for soil nutrients (e.g., SOM, TK, AP, and AK contents, **Table 2**), although these two crops did not exist at the same time (Duan et al., 2019). On the other hand, the root exudates of the two species are inconsistent, and the microbial communities absorbed and enriched are also different, resulting in changes in soil physical and chemical properties and soil structure (Li et al., 2014). Therefore, it affects the seed germination and plant growth of the same or heterogeneous plants, especially for heterogeneous plants (Lendzemo et al., 2009).

N is a key element in the formation of nutrient value of forage. With mowing time, AN in soil decreased continuously, which hindered the formation of CP in leaves and reduced the forage quality (**Table 2**; **Figure 1**). First, wheat is an important allelopathic crop and some substances (such as benzoxazinoids and polyphenols) in its root exudates can inhibit the growth of its neighboring heterologous species (Lendzemo et al., 2009; Hussain et al., 2022). The accumulation of these allelopathic substances in the soil may inhibit the growth of *C. esculentus*. Second, during the growth and development of plants, root exudates can not only affect the absorption and transport of nutrient availability in the soil, but also adsorb some heavy metal elements around the root system to affect the crop rotation next year (Sun et al., 2018).

Indeed, *C. esculentus* has been treated as a weed in many countries (Devries, 1991; Werle et al., 2022). Conversely, the rotation showed a positive effect on *C. esculentus* by enhancing nutrient (e.g., TN and TK) accumulation in tubers (**Figure 1D, F**). Tubers are a key reproductive organ of *C. esculentus* and its nutrient content determines the initial conditions of germination (Ozcan et al., 2021). Nutrient allocation to reproductive organs is an important stress adaptation strategy to increase the survival of offspring (Coello and Martinez-Barajas, 2016). Based on the above discussion, on the one hand, wheat should be carefully introduced into the rotation system of *C. esculentus*, while leguminous crops (e.g., Alfalfa and soybeans) may be preferred due to better nitrogen fixation and soil improvement ability. On the other hand, the application of organic manure is also a more effective way to overcome the obstacles caused by RC in this study by improving soil quality (Alburquerque et al., 2012).

### 4.2 Relationship between plant nutrient content and forage nutritive value and soil properties

Our study uncovered that soil pH is the vital factor influencing plant nutrient content and forage nutritive value (**Figure 4**). Previous studies have proved that pH can not only change the decomposition process of SOM and nutrient availability, but also directly affect the reaction rates of enzymes associated with biochemical reactions and soil microbial activities, thus it can regulate crop growth by affecting soil properties (Dick et al., 2000; Guo et al., 2010). The soil AK and sodium ion content are less, resulting in a relative excess of soil H^+^, leading to soil acidification (Dick et al., 2000; Ozcan et al., 2021). However, RC changed the soil pH value, making the soil TN, soil TP, soil TK, and soil available nutrients variation significantly, which affected the accumulation of nutrients and nutritional quality of the leaf and tuber (Guo et al., 2010). Higher soil pH was observed in RC systems than in CC (**Table 3**), that may be due to much higher uptake of nitrate (NO- 3) and compensatory secretion of OH^-^ by crops in the rotation system (Alvey et al., 2001). Higher level of Mg^2+^ may also contribute to the higher pH in RC system (**Table 3**). In our study, soil pH exerted a direct and indirect impact on plant nutrient accumulation and forage nutritive value by regulating the available nutrients, anions, cations, and total nutrients in soil (**Figure 4**). Therefore, a range of physical, chemical, and biological processes may be involved in the negative effects of rotations on nutrient accumulation and forage nutritive value. These processes will be explored by introducing key indicators such as plant physiology, microbial activity and soil enzyme activity in our future studies.

## 5 Conclusions

In conclusion, RC, irrespective of mowing time, exerted a positive effect on nutrient accumulation (e.g., total nitrogen, and potassium) in tubers, but a negative influence on nutrient accumulation (total nitrogen, phosphorus, and potassium) in leaves and roots, and their nutritive value (evaluated by CP, EE, ADF, and NDF). Soil properties, especially pH, may explain the variation of the performance of *C. esculentus* growing in CC and RC. The plant nutrient accumulation and forage nutritive value were directly affected by soil pH and were indirectly regulated by the variations in soil available nutrients, anions, cations, and total nutrients induced by soil pH. Therefore, RC for *C. esculentus* is not a good planting mode at any mowing time in terms of plant nutrient accumulation and forage nutritive value, likely due to degraded soil quality induced by nutrient competition between crops. For the intensive cultivation of *C. esculentus*, organic manure supplementation and rotation with legumes may overcome these adverse effects caused by rotation with wheat that will be explored in our future research.

## Data availability statement

The original contributions presented in the study are included in the article/supplementary material. Further inquiries can be directed to the corresponding authors.

## Author contributions

Conceptualization: YD, YZ, FZ. Data curation: YD, YZ and XC. Formal analysis: YD, YZ, and ZZ. Funding acquisition: FZ. Investigation: YD, YZ, XC, FZ. Methodology: YD, YZ and AU. Project administration: FZ. Resources: FZ. Software: YD, YZ and ZZ. Supervision: FZ. Validation: FZ, YD, YZ, WI and ZZ. Writing -original draft: YD, YZ. Writing - review and editing: YD, YZ, XL, FZ, WI, ZZ. All authors contributed to the article and approved the submitted version.
